# Multispecies entanglements in the virosphere: Rethinking the Anthropocene in light of the 2019 coronavirus outbreak

**DOI:** 10.1177/2053019620979326

**Published:** 2022-04

**Authors:** Anne Aronsson, Fynn Holm

**Affiliations:** 1 University of Zurich, Switzerland; 2 Institute of Asian and Oriental Studies, University of Zurich, Switzerland

**Keywords:** coronavirus, infectious diseases, multispecies entanglements, unusual encounters, virosphere

## Abstract

In this essay, we reevaluate the 2019 outbreak of a novel coronavirus (SARS-CoV-2) from the perspective of multispecies entanglements. It is argued that anthropogenic alterations in the biosphere will most likely accelerate the rate of multispecies pandemics in the Anthropocene. Using a textual analysis approach of anthropological and historical sources on the example of coronaviruses and live animal markets in China, we trace how the virosphere of wild animals from tropical regions comes into contact with the virosphere of humans and farmed animals in highly industrialized landscapes. We suggest that adopting a multispecies perspective on viruses can allow them to be understood as living processes that interact with other species in a realm called the virosphere. The rate at which novel infectious diseases are transmitted by bacteria and viruses has increased in recent decades. We argue that this is caused by side effects of the Anthropocene, such as deforestation, the surge in population growth and density, and anthropogenic climate change, which give rise to an increased number of unusual encounters between humans, nonhuman companion species, and wild animals. In this way, the virospheres of host organisms, which were formerly partly isolated, are allowed to converge and freely exchange infectious diseases, leading to a more homogenized virosphere. As anthropogenic alterations are set to continue in the future, we suggest that multispecies pandemics will likely increase in the following decades.

## Introduction

As the world has recently entered the epoch of the Anthropocene, we, as a species, are beginning to realize that successfully mitigating all side-effects of an anthropogenic biosphere, such as climate change and large-scale pollution, may be beyond our collective capacity. For example, the rates of novel infectious diseases have been increasing in recent decades, and despite health experts’ continuous warnings, such as those about the danger of multidrug-resistant bacteria, a worldwide pandemic was needed to bring these problems to the forefront of the political agenda. Global human society was not prepared for the novel coronavirus (SARS-CoV-2) outbreak in late 2019. However, we were not the only ones affected by the outbreak of the COVID-19 pandemic. SARS-CoV-2, like its close relative SARS-CoV and other coronaviruses, most likely originated in bats. As it can easily jump between human and nonhuman species, the COVID-19 pandemic is a multispecies event, and we might be the ones to blame.

This paper discusses how the Anthropocene may be accelerating the rate of cross-species infectious diseases caused by bacterial and viral pathogens, thereby increasing the risk and severity of multispecies pandemics. Using the example of coronaviruses, we discuss how anthropogenic alterations in the biosphere have accelerated unusual encounters between humans and nonhumans from ecosystems that were formerly partly isolated and led to the intermingling of their virospheres. We use the first coronavirus pandemic, which occurred in 2002 in Foshan, Guangdong, China, as an example to show how wildlife animals and their pathogens enter highly industrialized landscapes and intermingle with a new virosphere. As anthropogenic alterations are expected to continue in the future, we suggest that multispecies pandemics will likely increase in the following decades.

Our discussion of recent coronavirus outbreaks in the Anthropocene is situated within the academic field of multispecies ethnography. In contrast to animal studies that focus mainly on human–animal interactions, a multispecies approach is interested in the entanglements between all living beings in dynamic milieus. By exploring the sites of multispecies entanglements, ethnography offers important insights into the many consequential ways that “human nature is an interspecies relationship” (Tsing, 2012: 144). Driven in part by recent scientific findings, such as the role of microorganisms in the human body, multispecies ethnographic writing is increasingly focusing on the relationships and entanglements between humans and other species—animals, plants, and microbes—in cultural research.^1^ Some researchers extend the term “liveliness” to even chemical “species,” such as rocks or weather systems, while others are willing to include gods, ancestors, and spirits in this category (Van Dooren et al., 2016: 4). In general, a multispecies approach focuses on the many agents that bring one another into being through entangled relations (Van Dooren et al., 2016: 3).

Our multispecies analysis includes not only humans and nonhuman animals but also the pathogens they host in their bodies, particularly viruses. Humans and their close companion species have closely co-evolved with a large number of viruses, creating distinct virospheres. The recent increase in infectious diseases is partly caused by so-called unusual encounters with wild animals and their virospheres. As Stephanie Erev explained in her 2018 essay “What is it like to become a bat?” unusual encounters “are unusual in the sense that they bring familiar kinds of things together in unfamiliar and perhaps slightly unsettling ways” (Erev, 2018: 130). For example, the introduction of a species and its pathogens to a new habitat might be a key driver of the extinction of other species, or it might be the beginning of a new assemblage and co-evolutionary relationship.

Donna Haraway’s work underscores this multispecies interface and its relation to mutual ecologies. Exploration and awareness are developed at the level of the biotic landscape, where humans and animals coexist, through examination of the concept of social relationships and their impacts across all species (Haraway, 2003). Haraway uses the expression “becoming-with” to describe this phenomenon, arguing that humans can never exempt themselves from Earth’s ecological community. “Becoming with” reinforces the notion that all life on this planet is interconnected and that believing in human exceptionalism is foolish (Haraway, 2008). At the same time, while life is largely interconnected, it has always found niches and evolved partly in isolation to better adapt to different environments, resulting in great biodiversity. In the Anthropocene, human ecological alterations, such as deforestation, transhumance or agriculture, are simplifying many ecosystems, sometimes consciously and sometimes unconsciously. This is allowing countless species to cross formerly insurmountable geographical boundaries, reducing genetic isolation and overall biodiversity. This type of interconnectedness is a key feature of the Anthropocene, but one that might come at a great cost.

## Exploring the virosphere

Viruses are an especially interesting case of multispecies entanglements. It is now accepted that viruses are, by far, the most abundant organic entities we know of; in fact, they are probably more common than all other forms of life combined (Crawford, 2018). While many biologists claim that viruses do not fulfil all criteria for being alive, we can best understand them as living processes that exist and interact with hosts in a realm called the virosphere (Dupré and Guttinger, 2016; Suttle, 2005). The virosphere describes the parts of the biosphere where multispecies entanglement occurs between viruses and their unwilling hosts. Though viruses are generally regarded as parasites that cause diseases, they are also responsible for stabilizing countless ecosystems worldwide. For example, by killing 20%–40% of marine bacteria daily, they convert bacterial biomass into particulate and dissolved organic carbon, which other microbes can use as organic base material. This material would otherwise sink to the ocean floor, making it inaccessible to most lifeforms (Crawford, 2018: 17–20).

Every ecological niche in which life can be found has been penetrated by the virosphere. Over 100 million types of viruses infect all species of living beings, including animals, microbes, and plants. As these beings evolve over time, so do the viruses they carry, resulting in millions of years of co-evolution between biological life and viruses. The unique set of viruses that can be found inside a host organism is called a virome (Dupré and Guttinger, 2016). We suggest that a group of hosts with related viromes possesses their own specific virosphere that, depending on the circumstances (i.e. environmental and/or biological), may partially overlap with other virospheres or continue to develop in isolation.^2^

Specific virospheres have influenced the direction of human history, often in the form of infectious diseases, together with other pathogens such as bacteria and fungi. One prominent example is the collapse of indigenous societies and their near-complete replacement in the Americas when they came into contact with the virosphere of European settlers (Crosby, 1972). Human populations have always shared a virosphere, not only with the other human populations with which they came into contact but also with geographically proximal nonhuman animals. Not all virus infections are harmful to humans; some viruses in our body infect and kill harmful bacteria or train our immune systems to handle more deadly viruses, shutting out viral competition and enhancing the host’s survivability (Mathew, 2019).

Viruses cannot replicate themselves without the help of other cells. Free-floating virus particles, called virions, attach to proteins on the surface of potential host cells. Then, the virus hijacks the protein-making mechanisms of the host cells and forces them to produce thousands of new virions to be released into the organism (Bandea, 1983). Thus, from the perspective of a virus inside the virosphere, it is not the species, but the cell membranes and whether they can breached, that is relevant. In the known virosphere, over 70% of viruses infect only one or two host species; often, viruses cannot bond to the receptor protein on the cell surface of another potential host species (Rodrigues et al., 2017: 3). However, some viruses have a much wider range of hosts. For instance, many coronaviruses can infect a large number of mammals and avian species, as they can receive genomic fragments from other coronaviruses when they co-infect the same host. Through a process called recombination, novel strains of coronaviruses are created that might be able to cross the species barrier (Chan and Chan, 2013).

Like the ebolavirus or measles virus, coronaviruses are RNA viruses (in contrast to DNA viruses, which cause diseases like smallpox or herpes). They were first discovered in the 1960s, and so far, seven strains of coronaviruses are known to infect humans. Coronaviruses cause diseases in mammals and birds ranging from the common cold to severe pneumonia, in the cases of SARS, MERS, and COVID-19 (Li et al., 2020). As RNA viruses possess no proofreading mechanism for replicating in host cells, mutations occur very quickly. While most mutations are either inconsequential or even harmful to viruses, the few useful ones are essential for creating newer, faster, and more effective methods of infecting hosts without being wiped out by the host organism’s immune system. Sometimes, together with RNA recombination, these mutations allow viruses to cross the species boundary (Crawford, 2018: 10–12).^3^

## The virosphere in the Anthropocene

The term “Anthropocene” describes the large-scale anthropogenic alterations of the biosphere that can be detected in geological sediment and are changing the global climate (Purdy, 2015). Despite legitimate concern regarding the term’s usefulness and attempts to change the naming convention, the concept of the Anthropocene is mostly accepted in the humanities and natural sciences (Haraway, 2015; Latour, 2014; LeCain, 2015). However, the exacting starting point of the Anthropocene is a point of contention. It can be defined as the development of agriculture ten thousand years ago, after the discovery of America and the beginning of the Columbian exchange in 1492, the Industrial Revolution around 1750, or the Great Acceleration and worldwide atom bomb tests in the 1950s and 1960s (Lewis and Maslin, 2015; McNeill and Engelke, 2014). Even millennia before the human species became geological agents (Chakrabarty, 2009), human societies influenced and altered local environments and ecosystems. Human encroachment on the biosphere is a complex process that interacts with naturally occurring climate change and progresses at different speeds in different places (Chakrabarty, 2018; Ruddiman, 2005).

A factor that has heavily influenced the development and spread of the human race—and its companion species—has been infectious diseases caused by bacteria, viruses, and fungi. Environmental historians have long suggested that the hegemony of Western Europe over the rest of the world during the past 500 years was partly achieved by the spread of infectious diseases, such as smallpox, measles, and influenza. Additionally, certain endemic infectious diseases acted as barriers against European colonization in tropical region (Crosby, 1986). Therefore, from the perspective of multispecies pandemics, we believe that the Columbian Exchange functions as a good starting point for the Anthropocene.

In the 20th century, improved sanitation and the discovery and development of vaccines and antiviral drugs led to the belief that infectious diseases would become irrelevant to humans. However, a study from 2008 found that the rate of emerging infectious diseases has actually increased since the 1940s, with a preliminary peak in the 1980s (Jones et al., 2008). Sixty percent of these new infectious diseases are zoonoses, meaning that the pathogens were transmitted from nonhuman animals to humans.^4^ In total, over 70% of infectious diseases originated in wildlife, and the number has increased drastically in recent years. The authors of this study believe that the emergence of these diseases is the product of anthropogenic and demographic changes.^5^ Soon, 60% of the human population will be living in urban areas with high population densities, and it is estimated that by 2050, half of the world’s population will live in tropical environments, where diseases are much more likely to break out. In many societies, the population is aging rapidly, which increases the risk of sickness. Furthermore, the global network of air, land, and water transportation brings not only humans but also nonhuman animals and countless pathogens across the globe each day (Bloom et al., 2017).

A danger made evident by the recent 2019 coronavirus outbreak is the imminent threat of progressive homogenization of the virosphere (and other pathogens). While the virosphere has always been associated with all life on earth, factors like the population density of host organisms, geographical isolation, latitude, or rainfall have posed natural barriers to the exchange of viral strains and led to development of partially specific virospheres. However, due to anthropogenic changes in recent decades, many of these former boundaries are disappearing, giving viruses more opportunities to infect new hosts and converge into a more uniform virosphere. For example, the depletion of biodiversity has been linked to the rise of zoonotic pathogens, which are responsible for the extinction or near-extinction of wild animals such as the black-footed ferret or sharp snouted day frog (Cunningham et al., 2017).

In many developed countries, anthropogenic ecological alterations have caused a widespread loss of biodiversity, possibly foreshadowing a sixth extinction (Kolbert, 2014). These alterations have also led to the simplification of ecosystems, for example, by converting them into monocultures for agricultural purposes. Humans alone consume 25%–40% of the biosphere’s net primary production, with a heavy bias towards developed countries (Williams et al., 2015: 200). As a result, many species of both fauna and flora have lost their former habitats and expanded their range into new ecosystems, leading to encounters between species with no previous contact (Williams et al., 2015: 203). In addition to habitat loss, these formerly unusual encounters are caused by accidental or intentional movement of human companion species. Formerly wild animals have adapted to live on the fringes of these anthropogenic ecosystems, migrated to different regions, or disappeared altogether. Some nonhuman animals, such as rodents and many avian species, have successfully adapted, and can move between different ecosystems quite easily. Thus, they bear the greatest risk of transmitting and spreading new diseases, for example when they get accidentally infected by a new pathogen from host organisms in tropical regions and then bring the pathogen to humans or nonhumans residing in highly industrialized landscapes (Olival et al., 2017). Voluntary contact between humans and nonhuman animals is mostly limited to companion species, who still have a risk of transmitting viral diseases to humans when not properly immunized. Other domesticated animals only come into contact with humans in specialized professions, such as veterinarians, farmers, and animal processing workers, all of whom—at least in theory—have to follow strict hygienic procedures (Woo et al., 2006).

Nevertheless, we should not underestimate the threat that large-scale artificial environments pose to humans and nonhumans in terms of developing and transmitting infectious diseases, as such areas hold thousands or even millions of farm animals in cramped spaces, often with questionable hygienic standards (Chastel, 2004). These practices are responsible for many zoonotic diseases, for example, by creating multidrug-resistant bacteria (Lowe, 2010: 642). In addition, they increase the risk that diseases, such as foot-and-mouth disease (FMD), which had its most prominent outbreak in the UK in 2001, will spread quickly among farm animals. However, while FMD is a zoonotic disease, the possibility that it will occur in humans is limited due to the minimal contact between the larger human population in developed countries and infected farm animals (Bauer, 1997).

In industrialized landscapes, the emergence of new infectious diseases is closely related to population growth and density, but in other kinds of landscapes, such as tropical landscapes, the richness of wildlife is a much more significant factor, as contact between humans and wild animals is much more common (Jones et al., 2008). Indeed, the spread of zoonotic diseases is especially prevalent in places such as the tropical forests of Southeast Asia and Africa, where humans are continually encroaching upon wildlife habitats and coming into closer contact with various nonhuman species (Woo et al., 2006). The destruction of wild animal habitats in forests either directly through deforestation, animal hunting, and construction of anthropogenic landscapes or indirectly through the side-effects of climate change, forces wildlife to relocate to habitats closer to human settlements. Tropical animals are host to an almost inexhaustible number of unknown pathogens, most of which have not yet crossed the species boundary, as the animals have lived and evolved in isolation from other species. However, when contact between tropical animals and humans or domesticated animals increases, so does the likelihood that viruses will cross species boundaries and transmit new diseases, against which the newly infected hosts will have no immunity (Carlson et al., 2020).

As we indicated in this section, increased population density and anthropogenic climate change have caused shifts in the geographic range of wildlife and have led to encounters with novel species and the exchange of viruses among formerly isolated species in different anthropogenic landscapes. In the next section, we use the example of Chinese live animal markets to illustrate how wild animals with an unknown virosphere are entering industrialized landscapes and accelerating the development of new infectious diseases.

## Coronaviruses and live animal markets

For years, infectious disease experts have warned of the dangers of Chinese live animal markets (Webster, 2004; Woo et al., 2006), as they are areas where different human and nonhuman beings—and their virospheres—come together, creating an ideal environment for pathogens to flourish. Selling animals for consumption at markets is not a new concept, and it is not limited to Chinese culture. It seems likely that markets have played a crucial role in creating and distributing pathogens throughout human history.

Southern Chinese markets primarily sell produce, spices, and other perishable goods, often including fresh meats (sometimes, but not always, wildlife) and fish. They are called wet markets because the concrete floors are washed often because of the goods they sell.^6^ Because of the close contact between humans and nonhuman animals, these markets are prone to transmission and amplification of zoonotic diseases (Woo et al., 2006), many of which can spread rapidly through the respiratory system.^7^ Purchasing fresh foods is a significant element of Southern Chinese culture, as it is believed that freshly prepared meals made from live animals are tastier and more nutritious than those made from frozen foodstuffs. In addition, the region’s fondness for special delicacies has led Southern Chinese live markets to offer a great variety of illegally and semi-legally hunted wild animals. The popularity of meals prepared from live animals has led to live markets being located within residential areas for easy accessibility, which allows for frequent contact between the humans in those residential areas and live animals (Woo et al., 2006). Thus, these markets have remained not only significant economic establishments but also perfect incubation hubs for transferring viruses across species (Lynteris, 2016).

Wild animals from tropical forests, such as the pangolin, are especially popular in many Southeast and East Asian countries. These animals are often hunted and eaten in Vietnam and Laos, but sometimes they are brought to China via an expanding regional network (Bell et al., 2004). In this way, species from isolated tropical landscapes in Southeast Asia meet and intermingle with farm animals from highly industrialized landscapes in Northern China. Various animal species are kept alive in small cages stacked on top of each other in deplorable hygienic conditions, usually with significant shedding of animal excreta. Considering that vast quantities of excreta and blood allow for the free inter-species transfer of pathogens, live animal markets are unique zones for the transmission of zoonotic diseases to humans and homogenization of the virosphere. A particular virus strain might only be able to infect a certain animal species, but it is often only a matter of time and exposure before viruses intermingle with each other and exchange genetic information or develop completely new mutations, allowing them to eventually cross the species boundary (Woo et al., 2006).

Determining the factors that lead to the emergence of viral diseases is complex. Potential pathogens reside in areas that can be either biotic (e.g., nonhuman animals) or abiotic (e.g., soil). The occasional transmission of these pathogens to humans can lead to small, undetected, sporadic outbreaks, as in the case of SARS-CoV. The live animal market suspected of causing the SARS-CoV outbreak in November 2002 is situated in Foshan, Guangdong Province, in Southern China. A 2003 study found that 13% of animal traders who conducted business at this market had developed antibodies to the disease, although none had been previously diagnosed with SARS-CoV, compared with just 1%–3% in the control group (Centers for Disease Control and Prevention (CDC), 2003). In addition, samples from caged animals at a Shenzhen live animal market revealed that masked palm civets, raccoon dogs, and Chinese ferret badgers all tested positive for SARS-CoV (Guan et al., 2003). Subsequent studies have shown that a range of other animals at live animal markets and farms in China, including cats, red foxes, lesser ricefield rats, geese, wild boars, and pigs, also developed antibodies to SARS-CoV (Shi and Hu, 2008). Unlike most other viruses, coronaviruses typically easily cross the species boundary, as the spike protein of the virion that locks to a cell receptor is present in both humans and many animals that can be found at live animal markets (Sun et al., 2020b).

It was initially believed that masked palm civets were responsible for spreading the virus to humans as staff and customers contracted SARS-CoV after working and eating at a local restaurant in Guangdong Province where these animals were held in cages (Wang et al., 2020). Masked palm civets, which originally lived in mountains and hill forests far from humans, are indigenous to Southeast Asia and can also be found in Southern China. Wild civets have been caught and bred in farms for human consumption since the 1950s. In 2003, more than 40,000 of these animals were raised at 660 farms across China (Shi and Hu, 2008: 76). Sample testing revealed that most civets infected by SARS-CoV became infected not in the wild or on farms, but in live animal markets, although this evidence was not completely conclusive (Shi and Hu, 2008: 76); one research team indicated that the civets might have initially been infected by humans, not the other way around (Janies et al., 2008). In the case of SARS-CoV, determining whether humans, civets, or racoon dogs first contracted the virus is no longer possible (Woo et al., 2006).

In 2005, SARS-CoV was found in wild Chinese horseshoe bats^8^ living in a cave in Yunnan Province. Scientists have since concluded that these bats were the original hosts of many coronaviruses (To et al., 2013). Bats are the only mammals able to fly, and they represent the largest group of mammals, with more than 1,300 species (approximately 20% of all mammal species). Their bodies host hundreds of dangerous viruses, which are sometimes transferred to humans, including the ebolavirus, Marburg virus, and Nipah virus. Perhaps as a side effect of the evolutionary pressure for bats to develop a higher body temperature in order to fly, their immune systems developed differently from other mammals, and their cells appear less capable of detecting foreign DNA. This makes it easy for DNA viruses, such as herpesviruses, to infect them. Typically, a virus’s countermeasures against a host’s antiviral responses cause increased morbidity and mortality in the host organisms. However, bats’ ability to host many otherwise deadly viruses without exhibiting any symptoms suggests that viruses cannot deploy these countermeasures against bats, making them the perfect reservoir for zoonotic diseases caused by both RNA and DNA viruses (Banerjee et al., 2020).

In the Anthropocene, contact between humans, domesticated animals, and bats has increased significantly. Habitat fragmentation and the loss of, for example, forests and grasslands has led many bat species to migrate and adapt to urban and agricultural environments. This increases the risk of spillover from the bat virosphere (Voigt and Kingston, 2016). Other factors accelerating the rapid dissemination of the coronavirus include climate, weather, and seasonality. Extreme weather events, such as droughts, temporarily reduce the resilience of potential host species, while long-term climatic change may create new opportunities for viruses to spread to other global regions (Curseu et al., 2010). Some species, including migratory birds, rodents, bats, and, of course, humans, are especially likely to become “super-spreaders” of viral infections to new regions and species (Carlson et al., 2020). During the SARS-CoV outbreak in Foshan in November 2002, a massive drought was causing problems for local farmers. Likewise, in December 2019, Wuhan, the suspected origin of the SARS-CoV-2 outbreak, was experiencing the worst drought in the past 40 years.^9^ The recent rise in extreme droughts in China has been linked to poor environmental management and anthropogenic climate change (Jiang et al., 2015) as well as to the rise of viruses, as cold, dry conditions are conducive to virus survival and weaken humans’ resistance to viral infections. Moreover, wild animals are traditionally eaten during the winter months in Southern China because of several festive events during that time of the year, such as the Chinese New Year in February, leading to greater risk of exposure (Sun et al., 2020a).

As with SARS-CoV, bats are suspected to be the original bearers of SARS-CoV-2 (Lam et al., 2020). Early reports indicated that the SARS-CoV-2 outbreak in Wuhan, Hubei Province, in December 2019 might have originated near the Huanan Seafood Wholesale Market. However, at the time of writing, this has yet to be confirmed, and other possible outbreak scenarios are under consideration (Sun et al., 2020a).^10^ Eben Kirksey has rightfully pointed out that Western media coverage often portray old stereotypes when writing about the eating habits of Chinese people and the consumption of wild animals (Kirksey, 2020). However, the possible connection between the Huanan Seafood Wholesale Market and the 2019 novel coronavirus outbreak was not an invention of Western media; it was first reported on December 31, 2019, to the World Health Organization by Chinese authorities.^11^ Since then, reports have shown that the very first person infected with SARS-CoV-2 had not visited the seafood market, making the true origins of this novel coronavirus unclear at the present time. Nevertheless, by January 2, 2020, 41 patients in Wuhan hospitals were found to have the novel coronavirus, of whom 66% did have contact with the market (Huang et al., 2020). As the market was immediately closed after the first outbreak, scientists had no time to gather samples from the wild animals sold there. Subsequent research has shown that caged pangolins from a research organization and wild pangolins seized in an anti-smuggling operation between Malaysia and China hosted multiple coronavirus strains similar to SARS-CoV-2. This indicates that COVID-19 is a multispecies pandemic (Sun et al., 2020a).

Live animal markets are one example of how wild animals with an unknown virosphere, which has evolved partly in isolation, are brought into industrialized landscapes. In the cityscape of China, viruses not only find a high density of potential human and nonhuman hosts but also are provided with the right conditions to quickly spread around the globe. However, Chinese wet markets are not all bad, nor are they the sole source of cross-species viral infections. They are just one example of how multispecies entanglements in the virosphere can occur in the Anthropocene. Even permanent closure of live animal markets will not be able to prevent the increase in encounters between the shrinking number of remaining species that live in the wild and the consequences of bringing together partly isolated virospheres.

## Conclusion: Of (non)humans and viruses

The recent coronavirus outbreaks highlight the intermingling of human and nonhuman virospheres, which had developed in partial isolation. Previously, humans and animals lived much closer to one another, but while older forms of living together did cause disease outbreaks, they did not do so with the same frequency.

The constant movement of humans, nonhuman animals, and microbes—which is intensified by increased population density and anthropogenic climate change—has environmental implications across spaces and places, further entwining bodies, ecologies, and societies. If the current trajectory continues, eventually, most ecosystems in developed countries will become part of a largely unified anthropogenic landscape. In contemporary multispecies assemblages, viruses have become a threat to species survival: “The sense that past and present are tied to but do not contain the future for either humans or influenza viruses is inherent in an ontology of the multispecies cloud. Our futures lie at the junctures where forms of the human, animal, and microbe meet and where each sustains—and clouds —the limits and possibilities of the other” (Lowe, 2010: 645). In this scenario, wild animals will adapt to the anthropogenic landscape, bringing with them a great number of unknown viruses that will cause havoc not only for humans but also for their domesticated animals: “Identical viral genes have been found in vastly different habitats on opposite sides of the world, suggesting that sequences are constantly being copied and pasted from virus to virus around a global DNA superhighway” (Hamilton, 2008: 39). It is likely that the portion of the virosphere that humans and nonhuman animals share will further increase. A mutation of one virus strain will quickly transcend species boundaries and, potentially, evolve into a multispecies pandemic, leading to the development of increasingly complex and intertwined assemblages. Further research in the natural and social sciences is needed to reveal the role of social factors in the frequency of zoonoses.

Currently, our mode of being is dependent on complex entanglements with animals and ecosystems. In this essay, we examined the Anthropocene as a multispecies world in the process of being formed and the nature of humans as part of an interspecies relationship. The multispecies assemblages unleashed during the current pandemic bring together various species through the entanglement and blurring of boundaries between humans and foreign virospheres. Multispecies ethnography facilitates discussion of this topic, as it encourages social scientists to ask what happens when humans and their interspecies, multispecies, and living processes—in this case, viruses—with which they have relationships become increasingly entangled.

To conclude, the outbreak of three highly infectious diseases caused by coronaviruses—SARS, MERS, and COVID-19—in the span of only twenty years is no coincidence. The collective agency and impact of humanity have never been greater than in the Anthropocene. However, we lose this agency as the negative effects of the Anthropocene increase in severity, impact, and volume. While the dangers of anthropogenic climate change have become clearer for most of us, the 2019–2020 SARS-CoV-2 outbreak reveals a similarly grave danger to humanity. Globalization, in combination with the emergence of a single, interconnected global economy, affects almost all human and nonhuman spheres. Even the virosphere has become globalized, and the assemblages between organic species and abiotic actors grow exponentially (Haraway, 2015: 159). The point is brought home most effectively by Haraway, who claims that the Anthropocene has severe discontinuities—“what comes after will not be like what came before”—and our task is to make this time as short as possible and “cultivate with each other in every way imaginable epochs to come that can replenish refuge” (Haraway, 2015: 160).

## Notes

1. Haraway (2003), *The Companion Species Manifesto*; Das (2013), “Being Together with Animals”; Haraway (2014), “Speculative Fabulations for Technoculture’s Generations”; Helmreich et al. (2015), *Sounding the Limits of Life*; Kohn (2013), *How Forests Think: Toward an Anthropology Beyond the Human*; Lowe and Münster (2016), “The Viral Creep”; Kirksey (2014), *The Multispecies Salon*.

2. It is important to note that this viral process can travel wide and far. When this process is restricted this does not just have to do with the immune system but there are more basic molecular networks and interactions that restrict how the virus can move across the landscape of living entities or processes. It is not only related to whether the target has the matching surface receptors but also has to do with the internal machinery of the cell, for instance, the molecular systems it has to process RNA. As such, the virus cannot do much on its own and there are many compatibility issues that decide where the virus can go.

3. The wide variance in RNA viruses’ genetic code makes it difficult to separate them into clearly defined populations. Clusters of viruses with mutated genomes are called “viral clouds” or “viral quasispecies.” See Lauring and Andino (2010); Lowe (2010).

4. For more on the topic of zoonoses and pandemics, see Quammen (2012).

5. In more than half of emerging infectious disease events, bacteria and rickettsia are the responsible pathogens, and viral or prion pathogens are responsible for one-fourth of cases. See Jones et al. (2008: 991).

6. Personal conversation with Caroline Merrifield, 23 April 2020. See also Merrifield (2020).

7. Estimates suggest that SARS-CoV-2 is less deadly than SARS-CoV and MERS, but it has a higher infection rate than seasonal influenza; see Callaway et al. (2020).

8. To put human–bat interactions into perspective, we have to see humanity as a spatially, synchronically, and diachronically diverse entity. We hail from forests and caves, and interactions with bats have been the norm, see Hawkins et al. (2016). The authors argue that humans have been consuming bats for thousands of years, but today we are not as used to bats as in previous epochs. Regarding the co-evolution of humans, nonhuman animals, and viruses, the encounter with bats is not novel. This does not imply, however, that there is no level of isolation but we also need to consider that close contact is not entirely novel either.

9. Moreover, it appears that the combination of urbanization and air pollution exacerbates the transmission and severity of the disease; see van de Pas, “Deep Economic Integration and the Impact of Lockdowns.” See also Martelletti and Martelletti (2020).

10. We also need to consider that searching for the emergence of the coronavirus in the Hunan wet market might have been a strategic maneuver by the Chinese government since outbreaks disrupt the social order and society (see Keck, 2020). Condemning wet markets could be seen as a convenient way for China to evade social unrest, and closing a market is much easier than issuing travel restrictions and/or imposing complete lockdowns.

11. “WHO | Pneumonia of Unknown Cause—China.” The original Wuhan Municipal Health Commission briefing, which was in Mandarin, has been removed from the website, but it can still be accessed using the Wayback Machine, a digital archive of the World Wide Web (see WHO, 2020).
